# Earth’s evolving geodynamic regime recorded by titanium isotopes

**DOI:** 10.1038/s41586-023-06304-0

**Published:** 2023-07-26

**Authors:** Zhengbin Deng, Martin Schiller, Matthew G. Jackson, Marc-Alban Millet, Lu Pan, Katrine Nikolajsen, Nikitha S. Saji, Dongyang Huang, Martin Bizzarro

**Affiliations:** 1 Centre for Star and Planet Formation, Globe Institute, University of Copenhagen, Copenhagen, Denmark; 2 Deep Space Exploration Laboratory/CAS Key Laboratory of Crust-Mantle Materials and Environments, School of Earth and Space Sciences, University of Science and Technology of China, Hefei, China; 3 Department of Earth Science, University of California, Santa Barbara, Santa Barbara, CA USA; 4 School of Earth and Environmental Sciences, Cardiff University, Cardiff, UK; 5 Deep Space Exploration Laboratory/Laboratory of Seismology and Physics of Earth’s Interior, School of Earth and Space Sciences, University of Science and Technology of China, Hefei, China; 6 Institute of Geochemistry and Petrology, ETH Zürich, Zürich, Switzerland; 7 Institut de Physique du Globe de Paris, Université Paris Cité, Paris, France

**Keywords:** Geochemistry, Geodynamics, Geodynamics, Geochemistry

## Abstract

Earth’s mantle has a two-layered structure, with the upper and lower mantle domains separated by a seismic discontinuity at about 660 km (refs. ^1,2^). The extent of mass transfer between these mantle domains throughout Earth’s history is, however, poorly understood. Continental crust extraction results in Ti-stable isotopic fractionation, producing isotopically light melting residues^3–7^. Mantle recycling of these components can impart Ti isotope variability that is trackable in deep time. We report ultrahigh-precision ^49^Ti/^47^Ti ratios for chondrites, ancient terrestrial mantle-derived lavas ranging from 3.8 to 2.0 billion years ago (Ga) and modern ocean island basalts (OIBs). Our new Ti bulk silicate Earth (BSE) estimate based on chondrites is 0.052 ± 0.006‰ heavier than the modern upper mantle sampled by normal mid-ocean ridge basalts (N-MORBs). The ^49^Ti/^47^Ti ratio of Earth’s upper mantle was chondritic before 3.5 Ga and evolved to a N-MORB-like composition between approximately 3.5 and 2.7 Ga, establishing that more continental crust was extracted during this epoch. The +0.052 ± 0.006‰ offset between BSE and N-MORBs requires that <30% of Earth’s mantle equilibrated with recycled crustal material, implying limited mass exchange between the upper and lower mantle and, therefore, preservation of a primordial lower-mantle reservoir for most of Earth’s geologic history. Modern OIBs record variable ^49^Ti/^47^Ti ratios ranging from chondritic to N-MORBs compositions, indicating continuing disruption of Earth’s primordial mantle. Thus, modern-style plate tectonics with high mass transfer between the upper and lower mantle only represents a recent feature of Earth’s history.

## Abstract (web summary)

Titanium isotope measurements for chondrites, ancient terrestrial mantle-derived lavas and modern ocean island basalts imply the preservation of a primordial lower-mantle reservoir for most of Earth’s geologic history.

## Main

The accretion history of terrestrial planets is punctuated by a global magma ocean stage, which leads to planetary differentiation and the establishment of important reservoirs, such as core, mantle and crust. The subsequent evolution and modification of these reservoirs can substantially affect the thermal and geodynamic regimes of planets. On the basis of mineralogy, rheology and seismic velocity, it has been established that the structure of Earth’s mantle is layered with a principal seismic discontinuity at about 660 km separating the upper and lower mantle domains^1,2^. However, the extent to which mass transfer occurs within the mantle throughout geologic history remains highly debated. Seismic tomography data suggest that subducted slabs can penetrate into the lower mantle and, at the current rate of mass exchange, Earth’s primordial mantle is not predicted to survive after prolonged whole mantle-scale convection^8–10^. Meanwhile, studies based on noble gases^11–15^, as well as tungsten^16^ and neodymium^17^ isotopes, have suggested instead the existence of primordial mantle domains in the modern deep Earth. Although the preservation of a primordial lower-mantle reservoir over long geological timescales is debated^18,19^, some geodynamic models show that preservation of primordial mantle domains can occur in a modern-style, whole-mantle convection regime characterized by deep subduction^20^. Also, both numerical modelling and geological observations^21–25^ suggest that Earth’s convection regime and, hence, the style of slab subduction may have also evolved considerably through time as a consequence of change in the heat flux and heat transfer^25,26^. As such, a potential solution to the conundrum is that the high mass transfer between the upper and lower mantle inferred from seismic tomography is a relatively recent feature of Earth’s geologic history such that the primordial, less degassed lower-mantle reservoir has been undergoing disruption but is not yet fully destroyed^27^. This hypothesis has not been fully evaluated given the lack of an unambiguous geochemical tool that can faithfully trace mass exchange between mantle and crustal reservoirs in deep time.

The stable isotope geochemistry of the refractory lithophile element Ti is a new tracer that can potentially provide a historical record of mass-exchange processes between mantle and crustal reservoirs. The continental crust of Earth can be formed through partial melting of subducting slabs^28^ and/or thickened mafic crust^29,30^, which produces felsic melts. Such magmatic processes can result in notable Ti isotopic fractionation between the felsic silicate melts and the residue from this melt extraction, that is, melting residues^3–7^. By contrast, partial melting of mantle peridotites seemingly does not fractionate Ti isotopes^3,4^. In detail, the δ^49^Ti values (that is, the per mil deviation of the ^49^Ti/^47^Ti ratio relative to the OL-Ti standard) of Archaean tonalite–trondhjemite–granodiorite (TTG) rocks and Phanerozoic granites^5,7^, as well as those of evolved volcanic rocks^3,6,7,31,32^, can be up to +2.0‰ higher than that of oxide-undersaturated mafic/ultramafic rocks^3,4^. Thus, recycling of melting residues from extraction of continental crust through either delamination or subduction is predicted to generate mantle reservoirs with heterogeneous δ^49^Ti (ref. ^4^). Furthermore, the largely immobile nature of Ti allows for a thorough investigation of Ti-stable isotope composition of Archaean mantle-derived rocks despite alteration and metamorphism. However, the application of stable Ti isotopes to understand mantle–crust differentiation and crustal recycling processes is hampered by the scatter in the δ^49^Ti values of chondrite meteorites used to define the BSE reference value^33–35^, which is probably because of a combination of factors, such as sample heterogeneity, analytical biases and uncertainties, as well as imperfect correction for mass-independent nucleosynthetic effects on ^46^Ti (Methods).

## The δ^49^Ti value of BSE

We developed new analytical methods for ultrahigh-precision Ti isotope measurements using the next generation of multicollector inductively coupled plasma source mass spectrometers (the Neoma Multicollector ICP-MS). Our protocol allows for the concomitant determination of the mass-independent (±0.15 epsilon on the mass-bias-corrected ^50^Ti/^47^Ti ratio) and mass-dependent Ti-stable isotope composition (±0.010‰ for δ^49^Ti) of individual samples to ultrahigh precision (see details in Methods). Using this approach, we analysed 24 chondrite meteorites covering all of the main chondrite classes. Despite substantial variability in ε^50^Ti values (that is, the per ten thousand deviation of the mass-bias-corrected ^50^Ti/^47^Ti ratio relative to the OL-Ti standard) between the analysed chondrites, they return a restricted range of δ^49^Ti values that define a weighted mean of +0.053 ± 0.005‰ (2 s.e., *n* = 22), excluding one CV3 (NWA 2364) and one LL3 (Talbachat n’aït Isfoul) that are probably subject to sample heterogeneity (Methods), which represents a threefold improvement in precision relative to previous estimates^33–35^. The first important observation emerging from the new BSE estimate is that it is distinct from the composition of the modern depleted mantle as sampled by N-MORBs^3,4^ (δ^49^Ti = +0.001 ± 0.004‰, 2 s.e., *n* = 12) that are thought to represent the ‘depleted MORB mantle’.

## Earth’s mantle δ^49^Ti value in deep time

**Fig. 1:**
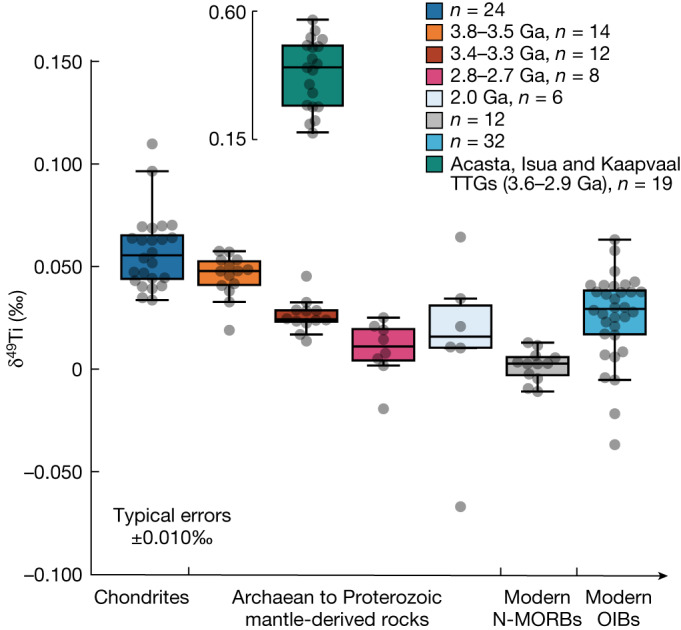
Mass-dependent Ti isotopic variations in bulk chondrites and Archaean, Proterozoic and modern terrestrial mantle-derived rocks from this study and the literature^3,4,6,7,31,32^. See the full dataset in the Extended Data Tables 1–5. Note that all the plotted terrestrial mantle-derived rocks are identified to be devoid of Fe–Ti oxide fractionation. The Archaean mantle-derived rocks from this study and ref. ^4^ have been arranged into three groups based on the formation ages (approximately 3.8–3.5 Ga, approximately 3.4–3.3 Ga and approximately 2.8–2.7 Ga), which show a progressive enrichment in the light Ti isotopes with age. The approximately 2.0 Ga data are from the Kangâmiut dykes samples (Southwest Greenland) in this study. Also shown are the data of approximately 3.6–2.9 Ga TTG rocks from this study and refs. ^5,7^ and that of modern N-MORBs from refs. ^3,4^. The box on each group of data defines the 25th–75th percentiles, with the medium value marked in the box and the whiskers standing for 0th to 100th percentiles, excluding outliers.

**Extended Data Fig. 1:**
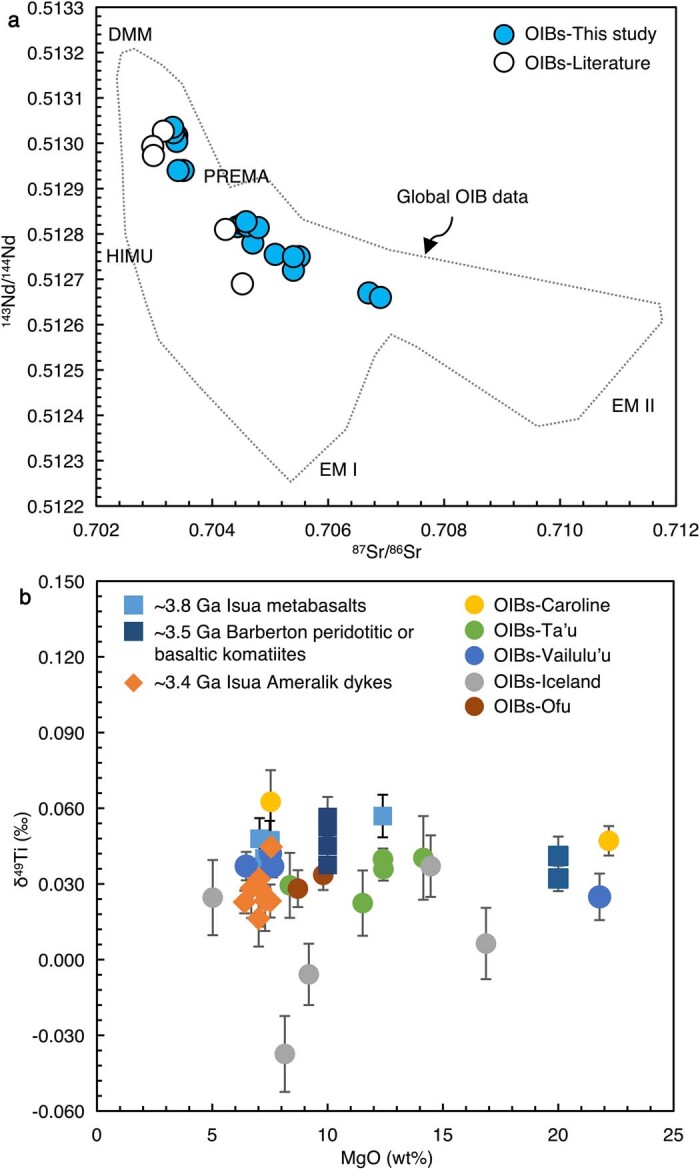
Chemical and isotopic data of the mantle-derived samples from this study. **a**, Strontium and neodymium isotope data of the studied modern OIB samples. The mantle end members and global OIB data are adapted from White^46^. **b**, Plot of δ^49^Ti versus MgO for the studied mantle-derived rocks. Note that the MgO contents of the studied approximately 3.5 Ga Barberton komatiites and those of basaltic komatiites are assumed to be 20 wt% and 10 wt%, respectively.

To better understand the importance of the lighter, non-chondritic Ti isotope composition of Earth’s modern depleted MORB mantle reservoirs, we analysed a set of terrestrial samples including 31 well-characterized Archaean to Proterozoic samples (one tonalitic and 30 mafic to ultramafic) with crystallization ages ranging from approximately 3.8 to approximately 2.0 Ga and 21 modern OIBs that have not experienced Fe–Ti oxide fractionation. The Archaean to Proterozoic samples include amphibolites (approximately 3.8 Ga), one Amitsoq gneiss (3.8–3.7 Ga), Ameralik dykes (approximately 3.4 Ga) and Kangâmiut dykes (approximately 2.0 Ga) from Southwest Greenland, as well as peridotitic to basaltic komatiites and tholeiitic basalts from the Kaapvaal Craton (approximately 3.48 Ga Komati Formation and approximately 3.33 Ga Kromberg Formation) and Munro Township from the Abitibi greenstone belt (approximately 2.7 Ga). The modern OIBs are from the Iceland, Caroline and Samoa hotspots, which have Sr and Nd isotope compositions spanning the curve defined by the depleted MORB mantle (DMM), the prevalent mantle (PREMA) and the enriched mantle type II (EM-II) (Extended Data Fig. 1a). As shown in Fig. 1, the early Archaean mantle-derived rocks have δ^49^Ti values indistinguishable from bulk chondrites, whereas the middle to late Archaean samples have progressively lighter compositions that extend towards the δ^49^Ti values of modern N-MORBs. By contrast, the approximately 2.0 Ga Kangâmiut dykes and modern OIB samples have highly variable δ^49^Ti values that range from the chondritic composition to values well below that of modern N-MORBs (Fig. 1). One approximately 3.8 Ga tonalitic sample (SD-2) from Isua records a high δ^49^Ti value of +0.205 ± 0.003‰, which is indistinguishable from previously reported δ^49^Ti values for TTG rocks from the Kaapvaal Craton and Acasta Gneiss Complex (δ^49^Ti = +0.173 ± 0.030‰ to +0.570 ± 0.030‰)^5,7^ (Fig. 1).

Our high-precision δ^49^Ti data for early Archaean komatiitic to basaltic rocks allows us to evaluate an earlier inference that partial melting of mantle peridotites on Earth produces only minor mass-dependent Ti isotopic fractionation^3,4^. Komatiitic magmas formed by about 25–40% partial melting of their mantle source^36,37^ and, as such, are expected to have extracted >90% Ti from their sources. By contrast, basaltic magmas that form from lower degrees of mantle partial melting (about 5–10%, for example, the approximately 3.8 Ga Isua pillow-textured metabasalts or approximately 3.48 Ga Barberton basaltic komatiites) extract approximately half of the Ti from their sources. Thus, a resolvable difference in δ^49^Ti is expected between the two types of magma if there is notable Ti isotopic fractionation between silicate melts and melting residues during partial melting of mantle peridotites. However, the comparable δ^49^Ti values between the approximately 3.8 Ga Isua metabasalts (+0.048 ± 0.005‰, 2 s.e., *n* = 5), the approximately 3.48 Ga Barberton komatiites (+0.044 ± 0.009‰, 2 s.e., *n* = 4) to basaltic komatiites (+0.048 ± 0.008‰, 2 s.e., *n* = 4) and chondrite meteorites (+0.053 ± 0.005‰, 2 s.e., *n* = 22) suggests that, in agreement with previous inferences based on various lines of evidence^3,4,38,39^, Ti isotopic fractionation between melts and residues from mantle partial melting is negligible. Thus, the near-zero Δ^49^Ti_melt-residue_ values inferred here suggest that metal-saturated melting with presence of Ti^3+^ is not relevant to the generation of terrestrial mafic/ultramafic magmas^40^. Moreover, the limited fractionation of Ti from mantle partial melting on Earth implied by our results supports the hypothesis that the studied mantle-derived rocks faithfully record the δ^49^Ti composition of their mantle sources. As such, our data suggest that sources of the studied mantle-derived rocks were characterized by chondritic δ^49^Ti values (δ^49^Ti = +0.053 ± 0.005‰) around approximately 3.8–3.5 Ga and evolved towards a modern depleted MORB mantle composition (δ^49^Ti = +0.001 ± 0.005‰) by approximately 2.7 Ga. This secular evolution is observed in both Southwest Greenland and the Kaapvaal Craton and is in line with the lower δ^49^Ti values observed in the late Archaean mantle-derived rocks from Belingwe, Yilgarn and Abitibi. By comparison, the approximately 2.0 Ga Kangâmiut dykes and modern OIBs were derived from the mantle sources different from the modern depleted MORB mantle reservoir.

## A long-lived primordial lower mantle

**Fig. 2:**
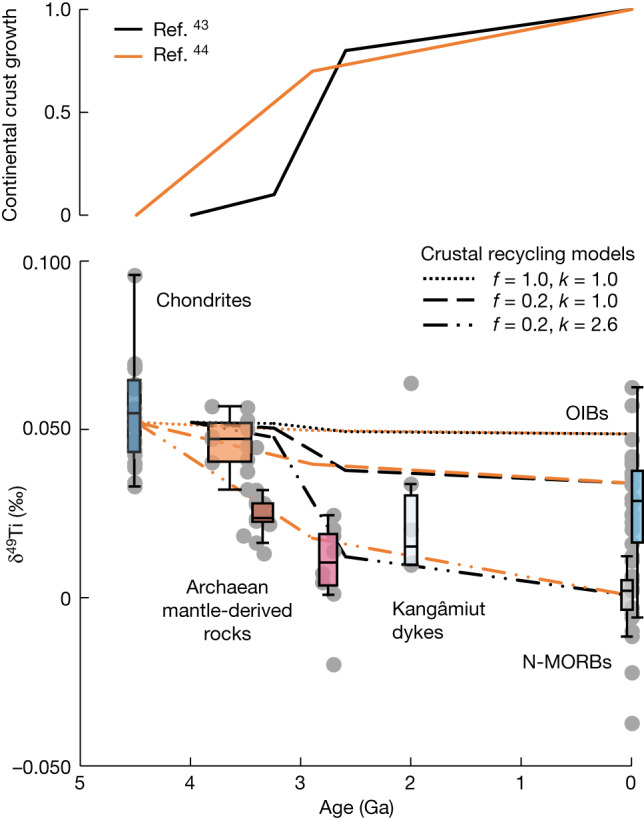
Continental crust extraction and the evolution of Ti isotopic composition in mantle-derived rocks. Chondrites and terrestrial mantle-derived rocks are shown in groups as defined in Fig. 1, with individual data points plotted as grey dots. The continental crust growth models from Taylor and McLennan^43^ and Dhuime et al.^44^ are shown on the upper plot. Crustal recycling models were made to quantify the potential Ti isotopic effects in the mantle from continental crust formation, in which *f* represents the fraction of Earth’s mantle to equilibrate with recycling crustal melting residues and *k* stands for the total mass of continental crust ever produced throughout geologic history after normalization onto its present mass (that is, about 0.55% of the BSE). See equations (13) and (14) and the related descriptions in Methods for details of the models. The box on each group of data defines the 25th–75th percentiles, with the medium value marked in the box and the whiskers standing for 0th to 100th percentiles, excluding outliers.

As indicated by the heavy Ti isotopic composition of Archaean TTGs, Phanerozoic granites and differentiated volcanic rocks^3,5–7,31,32^, the formation of a felsic continental crust results in the production of an isotopically light crustal melting residue. Thus, we interpret the observed secular change in the Ti isotopic composition of the Archaean mantle as evidence for the progressive recycling of melting residues through delamination or subduction to Earth’s mantle following continental crust extraction^41,42^, requiring full isotopic equilibration between the mantle reservoir and the admixed melting residues. Notably, the observed shift towards lower δ^49^Ti values in the source of Archaean mantle-derived rocks between approximately 3.5 and 2.7 Ga coincides with the main epoch of continental crust extraction proposed in previous studies^43,44^ (Fig. 2). Adopting the current mass of continental crust (that is, about 0.55% of the BSE), neither whole-mantle convection nor layered-mantle convection with limited mass transfer between the upper and lower mantle can reproduce the approximately 0.052‰ fractionation in the mantle by recycling of melting residues from continental crust formation (Fig. 2). However, it is possible to generate the δ^49^Ti effect of about 0.052‰ through layered-mantle convection with limited mass transfer between the upper and lower mantle only if the mass of continental crust produced over geological time is greater than the current mass, namely, about 1.43% of the BSE. Such a high production of continental crust throughout Earth’s history has also been inferred in the recent continental crust growth models^45^, based on the integration of various proxies, such as neodymium and hafnium isotopes. This consistency between studies using distinct geochemical tracers suggests that the mass of continental crust produced in Earth’s history probably exceeded its present-day value, and a large portion of this crust has been destroyed and recycled into the mantle, meaning that a high mass of ancient continental crust has been stored in the deep mantle. An important finding of this work is that only a small fraction of Earth’s mantle (about 20%) has equilibrated with melting residues from continental crust extraction, implying limited mass transfer between the upper and lower mantle in the Archaean. Irrespective of an apparent separation of the lower and upper mantle, after the Archaean, some upwelling of primordial material from the lower mantle has probably occurred since 2 Ga, as evidenced by the elevated δ^49^Ti values in some of the studied approximately 2.0 Ga Kangâmiut dykes and modern OIBs (Fig. 2).

**Fig. 3:**
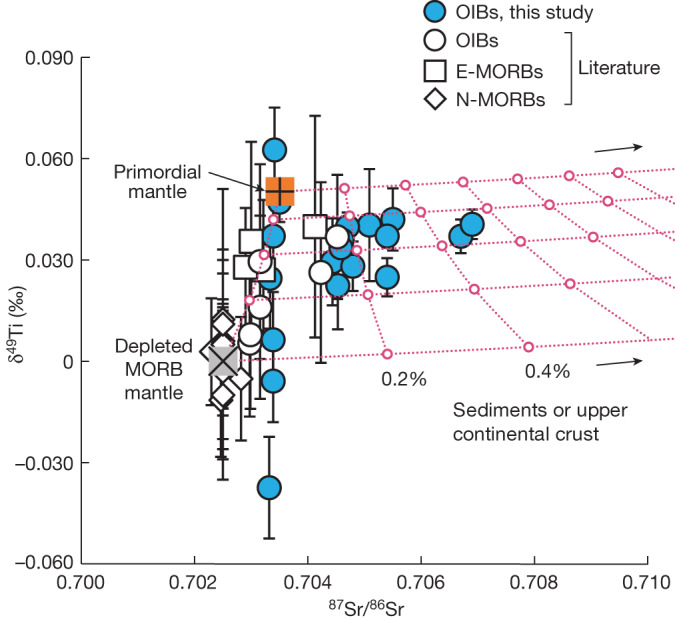
Sampling of a primordial mantle reservoir by mantle plume as evidenced by Ti and Sr isotopic records of the modern OIBs from the Iceland, Samoa and Caroline hotspots. Data of the OIB samples from Cape Verde and Azores in ref. ^3^ are shown as white circles. The N-MORB and E-MORB samples from refs. ^3,4^ are shown, for which the N-MORB samples without available Sr isotope data have been assumed to have ^87^Sr/^86^Sr = 0.7025. The dotted pink trajectories describe the effects from mixing in increments of 0.2% the ancient marine sediments or continental crust material with δ^49^Ti = +0.200‰ (refs. ^5,6^) and ^87^Sr/^86^Sr = 0.740 (ref. ^47^) into a modern depleted MORB mantle source with ^87^Sr/^86^Sr = 0.7025 (ref. ^63^) and δ^49^Ti = +0.001‰ (refs. ^3,4^) or into a mantle source with ^87^Sr/^86^Sr = 0.7035 and a primordial mantle δ^49^Ti of +0.052‰. Addition of recycled melting residues would lead to lower δ^49^Ti values in N-MORBs and some of the OIBs.

Modern OIBs, which are thought to sample a deeper mantle reservoir than MORBs^46^, provide an opportunity to explore the possible survival of a primordial material in the lower mantle. Although the sources of modern OIBs and enriched-MORBs (E-MORBs) record large δ^49^Ti variability, most have δ^49^Ti values that are 0.030–0.045‰ heavier than the composition of the modern depleted MORB mantle sampled by N-MORBs (Fig. 3). Given that marine sediments since the Archaean constantly record high δ^49^Ti values (+0.20‰ on average^5,6^), it is possible that the elevated δ^49^Ti signature of the OIB sources is a result of admixing of subducted marine sediments or, alternatively, upper continental crust material into a modern depleted MORB mantle reservoir. However, increasing the δ^49^Ti value of a hybrid mantle source by 0.030–0.045‰ using this process should also lead to highly radiogenic Sr isotopic signatures from sediments or upper continental crust^47^ in the OIB lavas, which is not observed here, except for some lavas from the Samoan hotspot showing ^87^Sr/^86^Sr ratios extending towards EM-II (Extended Data Fig. 1a). Thus, the predominantly heavy δ^49^Ti values of modern OIBs requires sampling of a mantle source that did not equilibrate with recycled crustal melting residues, which we infer to represent a primordial lower-mantle reservoir underlying the modern depleted MORB mantle (Fig. 3). Nonetheless, δ^49^Ti heterogeneity exists within the mantle sources of OIBs. The lower δ^49^Ti values relative to the chondritic composition seem to provide evidence for injection of not only recycled sediments or upper continental crust but also melting residues into the lower primordial mantle reservoir. We note that recycling of ancient restites from protocrust extraction in the sources of some OIBs has been suggested on the basis of tungsten isotopes^42^. This introduction of recycled material to the lower mantle traced by Ti isotopes is consistent with seismic tomography of the Earth that suggests a high rate of mass exchange between the upper and lower mantle in modern times^8,9^ and other geochemical evidence that indicates the presence of ancient subducted oceanic lithosphere in the sources of OIBs^47–50^. It has also been proposed that the anomalous noble gas and tungsten isotope signals in modern OIBs may reflect interaction with core material as opposed to sampling of a primordial mantle reservoir^51–53^. The refractory and lithophile nature of Ti makes its stable isotope composition impervious to the interaction with core material. Thus, the Ti isotope data reported here coupled with the noble gas and tungsten isotope signals identified in modern OIBs^11–16,54,55^ are most consistent with the survival of a primordial, less degassed mantle reservoir in the modern deep Earth.

## The evolving geodynamic regime of Earth

Our new Ti isotope data, which require limited mass exchange between the upper and lower mantle over a substantial part of Earth’s geologic history, provide new insights into Earth’s geodynamic evolution. The chondritic or primordial mantle-like δ^49^Ti value of the approximately 3.8–3.5 Ga upper mantle indicates limited production of felsic continental crust and recycling of melting residues during the early Archaean, pointing to a long residence of the primordial crust on Earth’s surface. By contrast, progressive enrichment of light Ti isotopes in mantle-derived rocks between approximately 3.5 and 2.7 Ga requires an acceleration in the growth of felsic continental crust and the recycling of melting residues into the mantle. The increased rate of crustal production and recycling suggest Earth’s transition into a geodynamic regime that allowed for progressive recycling of crustal materials back into the mantle. This is in line with the progressive homogenization of ^142^Nd variations preserved in rocks from the same time period^56,57^. Felsic continental crust can be generated without plate tectonics through partial melting of hydrated basalts at the base of a thickened crust^29,30,41,58,59^ or, alternatively, associated with a tectonic regime that includes active subduction of surface materials^28,60,61^. Regardless, the secular evolution of δ^49^Ti recorded by the Archaean mantle-derived lavas is best understood to reflect a transition in Earth geodynamic regime promoting accelerated crustal recycling around 3.5 Ga.

The mass of the mantle inferred to have equilibrated with the recycled melting residues (<30%) to explain the shift in Ti isotope composition in mantle-derived rocks is broadly consistent with that of the mantle located above the seismic discontinuity at about 660 km, suggesting that the phase transition associated with this discontinuity may have impeded mass exchange. Such a mantle separation is distinct from modern-style plate tectonics that are characterized by deep plate subduction and penetration of subducted slabs into the lower mantle. This may indicate that, unlike the modern-style regime, the subducted slab may have different fates in deep time, which may experience frequent slab breakoff under the temperature, composition and H_2_O conditions of the Archaean upper mantle^25,26^ or, alternatively, accumulate at the transition zone, at which density contrast between subducted slab and surrounding mantle reverses notably^62^. Thus, before 2.7 Ga, recycling and admixing of subducted slabs into the ambient mantle was limited to the highly convective upper-mantle region instead of penetrating through the mantle transition zone. The coexistence of primordial and evolved δ^49^Ti signals in modern OIBs and MORBs, respectively, requires that the transition between a layered and whole-mantle convective regime occurred late in Earth’s history. Thus, these results give credence to theoretical models suggesting that modern plate tectonics with deep slab penetration represents a transient phase in the evolution of planets^23,27^.

Finally, whereas the fundamental causes for the acceleration in continental crust growth and crustal recycling between 3.5 and 2.7 Ga remain unclear, our new δ^49^Ti data require a regime of mantle convection with limited mass transfer between the upper and lower mantle for a substantial part of Earth’s history. A possibility is that this epoch represents the onset of a tectonic regime allowing the subduction of plates or, alternatively, frequent crustal thickening, which—in both cases—will result in partial melting and extraction of felsic continental crust. Regardless, our data require that efficient recycling and homogenization of the melting residues from felsic continental crust generation was limited to the upper mantle, which implies the long-term preservation of a primordial lower-mantle reservoir. However, the highly variable Ti isotope compositions recorded by modern OIBs suggest that the primordial lower-mantle reservoir is undergoing disruption. Thus, modern-style plate tectonics with whole-mantle-scale convection and deep penetration of subducted slabs may only represent a transient and recent feature of Earth’s history.

## Methods

### Samples

The chondrite samples analysed in this study include one CI (Orgueil), two CV (NWA 2364 and Allende CAI-free matrix), six CM (Cold Bokkeveld, Murray, Murchison, Bells, Maribo and NWA 4428), two CO (NWA 1232 and NWA 763), one CH (SaU 290), two CK (NWA 1559 and NWA 1563), four CR (NWA 530, NWA 1180, NWA 6043 and NWA 801), one EH (SaH 97159), three L (NWA 5697, Bovedy and Hedjaz) and two LL (Ragland and Talbachat n’aït Isfoul).

The Archaean to Proterozoic samples from three locations were also studied, comprising: (1) five approximately 3.8 Ga pillow-textured metabasalt/metagabbro samples (PB-1, PB-2, PB-3, GB-1 and MG-1), one approximately 3.8 Ga Amitsoq gneiss (SD-2), eight approximately 3.4 Ga doleritic samples of the Ameralik dyke swarm (AM-1, AM-2, AM-8, AM-9, AM-10, AM-12, AM-14 and AM-16) and six approximately 2.0 Ga Kangâmiut dyke samples (430931, 430970, 430981, 430988, 432108 and 432122) from Southwest Greenland, (2) two approximately 3.48 Ga komatiite (1973-543 and 1973-547) and four approximately 3.48 Ga basaltic komatiite samples (1973-544, 1973-545, 1973-546 and 1973-730) of the Komati Formation, as well as three approximately 3.33 Ga tholeiitic basalt samples (1973-549, 1973-555 and 1973-733) of the Kromberg Formation from the Kaapvaal Craton in South Africa, and (3) three approximately 2.7 Ga Pyke Hill komatiite samples (1990-63, 1990-65 and 1990-67) in Munro Township from the Abitibi greenstone belt in Canada. The approximately 3.8 Ga Isua metabasalts and the approximately 3.45 Ga Ameralik dyke samples have been shown to have positive ^142^Nd excesses of +10.5 ± 0.7 and +4.9 ± 0.5, respectively^57^. The reduced ^142^Nd excesses in the Ameralik dyke samples relative to the older metabasalts have been attributed to a recycling of Earth’s primordial crust into the upper mantle^57^.

As well as the chondrite and Archaean/Proterozoic samples, we selected 21 modern OIBs for study, comprising: (1) ICE-14-16, ICE-14-18, ICE-12-27, ICE-14-29, ICE-14-32A and 408616 from the Iceland hotspot^54^, (2) KOS-13-4 and KOS-13-19 from the Caroline hotspot^64^ and (3) OFU-04-05, OFU-05-01 and OFU-05-18 of Ofu Island^65^, T16, T30, T33, T44 and T45 of Ta‘ū Island^65^ and AVON3-63-2, AVON3-70-9, AVON3-71-22, AVON3-73-1 and AVON3-77-1 of Vailulu‘u Island^66^ from the Samoa hotspot. Most of the analysed modern OIB samples have been characterized for both chemical (major and trace elements) and radiogenic isotope (Sr–Nd–Pb–He–W) compositions in the literature^54,55,64–66^. Most of the analysed OIB samples have higher ^3^He/^4^He ratios (up to 38.7 Ra, in which Ra represents a normalization onto the ^3^He/^4^He ratio of atmosphere) compared with that of N-MORBs (about 8 Ra)^54,64–66^. These OIB samples also have resolvable negative u^182^W values down to −13.8 ± 3.3 ppm (refs. ^16,55^).

Although fractional crystallization of Fe–Ti oxides can quickly lead to increasing δ^49^Ti values for evolved mafic lavas^3,6,7,31,32^, we argue that the mantle-derived rocks in this study are devoid of Fe–Ti oxide fractionation based on two observations: (1) although at fayalite–magnetite–quartz buffer Fe–Ti oxides normally start crystallizing at late stage of magma differentiation^67^ (MgO < 5 wt%), the measured samples have high MgO contents of >5.80 wt%, except for sample ICE-14-16 with MgO = 5.02 wt%, and (2) the lavas from the same age groups or the same oceanic islands did not show resolvable increase in δ^49^Ti with decreasing MgO contents (Extended Data Fig. 1b). We also note that some OIB samples contain the earlier crystallized olivine phenocrysts that would lead to much higher MgO contents, which—however—should have negligible effects on the Ti isotopic compositions of the studied samples in a whole-rock-scale owing to the low TiO_2_ contents in olivine.

### Sample dissolution and chromatographic purification of Ti

Powders of samples were weighed into precleaned Savillex beakers and dissolved with mixtures of 22 M HF and 14 M HNO_3_ acids in a 2:1 volume ratio. The modern OIBs and four reference materials (that is, BHVO-2, BCR-2, AGV-2 and BIR-1) were digested on a hot plate at 120 °C for four days. Note that all chondrite and Archaean ultramafic/mafic rock samples were digested in Parr bomb vessels at 220 °C for three days to ensure full dissolution of refractory phases. Dissolution of the dried samples in 5–10 ml 6 M HCl at 120 °C and evaporation was carried out several times to decompose the fluorides formed from HF digestion until clear solutions were obtained. An aliquot of each sample was taken and spiked with a prepared ^47^Ti–^49^Ti double spike to determine in advance the Ti concentration using an iCAP RQ inductively coupled plasma mass spectrometer at the Centre for Star and Planet Formation (StarPlan) at the University of Copenhagen. Afterwards, aliquots containing 6 µg Ti were taken and mixed with a ^47^Ti–^49^Ti double spike as described previously in ref. ^34^. The dried mixtures were dissolved with 6 M HCl at 120 °C overnight to ensure sample–spike equilibration.

Titanium was separated from matrix elements following a three-step purification protocol using AG1x8 (200–400 meshes) and DGA resins^34,68^, that is, first to separate Fe with 6 M HCl elution on AG1x8 columns, second to remove most of the major and trace elements through 12 M HNO_3_ elution and to collect Ti with Milli-Q H_2_O on DGA columns and third to purify Ti from the remaining matrix elements with 4 M HF cleaning on AG1x8 columns. An extra DGA pass can be carried out to remove trace amounts of Ca and Cr in the final Ti cuts. To destroy the resin particles and organics from column chemistry, the Ti cuts were treated with 14 M HNO_3_ at 120 °C before storage in 0.5 M HNO_3_ + 0.01 M HF acids.

### Neoma Multicollector ICP-MS

Titanium isotopic compositions of the purified samples were measured using the ThermoFisher Scientific Neoma Multicollector ICP-MS. Sample solutions with 500–800 ppb Ti dissolved in 0.5 M HNO_3_ + 0.01 M HF were introduced into the multicollector inductively coupled plasma source mass spectrometer by means of an APEX HF desolvating nebulizer from Elemental Scientific and a sapphire injector was used instead of the quartz-made injector to reduce the production of silicon fluorides from the use of HF solvent. An actively cooled membrane desolvation component was attached after the APEX to suppress oxide formation and to stabilize the signals, and N_2_ gas at a flow rate of a few ml min^−1^ was added to improve the sensitivity. Such a setting typically provides an intensity of around 15 V on ^48^Ti^+^ at an uptake rate of about 50 μl min^−1^ for a 600-ppb Ti solution under a medium mass-resolution mode.

The increased mass dispersion of the Neoma relative to earlier-generation instruments allows for a simultaneous monitoring of ^43^Ca^+^ (L5), ^44^Ca^+^ (L4), ^46^Ti^+^ (L3), ^47^Ti^+^ (L1), ^48^Ti^+^ (C), ^49^Ti^+^ (H1), ^50^Ti^+^ (H2), ^51^V^+^ (H3), ^52^Cr^+^ (H4) and ^53^Cr^+^ (H5) species in a single collector configuration. The medium mass-resolution mode on the Neoma (that is, *M*/Δ*M* ≈ 7,000) can resolve the main molecular isobaric interferences on the measured masses (for example, ^28^Si^16^O^+^ on ^44^Ca^+^, ^28^Si^19^F^+^ on ^47^Ti^+^ and ^36^Ar^14^N^+^ on ^50^Ti^+^). Measuring intensities on ^44^Ca^+^, ^51^V^+^ and ^53^Cr^+^ with those of Ti allows for a high-precision correction of the related isobaric interferences. To account for instrumental mass bias on the measurements from different sessions, a strict standard-sample bracketing protocol was used for all the multicollector inductively coupled plasma source mass spectrometer sessions in this study, that is, to analyse the OL-Ti standard solution before and after every sample analysis. Each analysis of the standard or samples comprises 100 cycles with 8 s integration time. On-peak zeros were measured before each sample/standard analysis in the same 0.5 M HNO_3_ + 0.01 M HF solution used to dissolve the sample/standard for 75 cycles with 8 s integration time. The typical background for the measurements is about 2–4 mV on ^48^Ti^+^. To evaluate data reproducibility, each sample has been normally analysed 4–8 times and four reference materials (that is, BHVO-2, BCR-2, AGV-2 and BIR-1) have been processed several times in parallel with the unknown samples.

### Concomitant derivation of Ti-stable isotope composition and nucleosynthetic component from double-spike measurements

An accurate determination of the Ti-stable isotope composition in meteoritic samples through a double-spike technique requires knowledge of the nucleosynthetic composition of the samples for correction. In the past, a separate protocol was needed for measurements of Ti nucleosynthetic components, that is, to analyse the samples purified without introducing a spike^68–70^. Because this approach is time consuming, previous Ti isotope studies^33,34^ have relied on literature values of the same meteorites or the same meteorite groups for correction. However, this is not ideal, as it can introduce artefacts on the Ti-stable isotope composition if discrepancies in the Ti nucleosynthetic component exist between the new digestion aliquots of meteorites and those in the literature.

It is, however, noteworthy that, after normalization onto the ^49^Ti/^47^Ti ratio, meteorites in bulk exhibit anomalies mainly on ^46^Ti and ^50^Ti (refs. ^69,70^), which are correlated following a relation of ε^46^Ti = (0.184 ± 0.007) × ε^50^Ti + (0.025 ± 0.009) (ref. ^71^), in which an epsilon notation is used to describe the magnitude of these isotopic anomalies. In this case, it is possible to derive both the Ti-stable isotope composition and the nucleosynthetic component in samples from the measured results of a sample–spike mixture by means of the following procedures, with the standard composition (that is, ${{\rm{R}}}_{{\rm{standard}}}^{46/47}$, ${{\rm{R}}}_{{\rm{standard}}}^{48/47}$, ${{\rm{R}}}_{{\rm{standard}}}^{49/47}$ and ${{\rm{R}}}_{{\rm{standard}}}^{50/47}$) and the ^47^Ti–^49^Ti double-spike composition (that is, ${{\rm{R}}}_{{\rm{spike}}}^{46/47}$, ${{\rm{R}}}_{{\rm{spike}}}^{48/47}$, ${{\rm{R}}}_{{\rm{spike}}}^{49/47}$ and ${{\rm{R}}}_{{\rm{spike}}}^{50/47}$) calibrated in advance:

1. The interference-corrected ^46^Ti/^47^Ti, ^48^Ti/^47^Ti and ^49^Ti/^47^Ti ratios from an analysis of either OL-Ti standard or unknown samples can be used for a primary double-spike inversion to obtain solutions for the three unknowns *λ* (that is, the proportion of ^47^Ti from the ^47^Ti–^49^Ti double spike in the sample–spike mixture), *α* (that is, the natural mass fractionation factor) and *β* (that is, the instrumental mass fractionation factor), as defined in a set of three non-linear equations^72^:
  1 $${F}_{i}(\lambda ,\alpha ,\beta ,n,m,T)=\lambda {T}_{i}+(1-\lambda ){n}_{i}{{\rm{e}}}^{-\alpha {P}_{i}}-{m}_{i}{{\rm{e}}}^{-\alpha {P}_{i}}=0,$$
  in which *n*, *m* and *T* represent the standard, the sample–spike mixture and the ^47^Ti–^49^Ti double spike, respectively, and each of them further comprises three known or measured Ti isotopic ratios (that is, ^46^Ti/^47^Ti, ^48^Ti/^47^Ti and ^49^Ti/^47^Ti), and *P*_*i*_ stands for a natural log of the atomic masses included in the selected isotope ratio *i*, for example, *P*_1_ = ln(45.9526316/46.9517631) for the ^46^Ti/^47^Ti ratio.
2. The ^50^Ti/^47^Ti ratio of the sample (${{\rm{R}}}_{{\rm{sample}}}^{50/47}$) can be derived from the measured ^50^Ti/^47^Ti ratio of the mixture (${{\rm{R}}}_{{\rm{mixture}}}^{50/47}$) and that of the ^47^Ti–^49^Ti double spike (${{\rm{R}}}_{{\rm{spike}}}^{50/47}$) using the defined *λ* and *β* values:
  2 $${{\rm{R}}}_{{\rm{sample}}}^{50/47}=\left[{{\rm{R}}}_{{\rm{mixture}}}^{50/47}\times {{\rm{e}}}^{-\beta \times {\rm{ln}}\left({m}_{50}/{m}_{47}\right)}-\lambda \times {{\rm{R}}}_{{\rm{spike}}}^{50/47}\right]/(1-\lambda ).$$
3. Afterwards, in the case that instrumental mass bias follows the exponential mass fractionation law as assumed in equations (1) and (2), deviation of the ^50^Ti/^47^Ti ratio of sample (${{\rm{R}}}_{{\rm{sample}}}^{50/47}$) from that of the standard composition (${{\rm{R}}}_{{\rm{standard}}}^{50/47}$) would be a combined result of the isotopic anomaly on ^50^Ti and the mass-dependent isotopic fractionation from natural processes, for which the magnitude of the latter can be quantified from the *α* value of the sample for correction. In this case, the ^50^Ti anomaly of the sample in an epsilon notation (ε^50^Ti) would be the same as the preliminary calculated values (that is, ε^50^Ti_prelim_):
  3 $${\varepsilon }^{50}{{\rm{T}}{\rm{i}}}_{{\rm{p}}{\rm{r}}{\rm{e}}{\rm{l}}{\rm{i}}{\rm{m}}}=[{{\rm{R}}}_{{\rm{s}}{\rm{a}}{\rm{m}}{\rm{p}}{\rm{l}}{\rm{e}}}^{50/47}\times {{\rm{e}}}^{-\alpha \times {\rm{l}}{\rm{n}}({m}_{50}/{m}_{47})}/{{\rm{R}}}_{{\rm{s}}{\rm{t}}{\rm{a}}{\rm{n}}{\rm{d}}{\rm{a}}{\rm{r}}{\rm{d}}}^{50/47}-1]\times \mathrm{10,000}.$$
  in which *m*_47_ and *m*_50_ stand for the atomic masses of ^47^Ti and ^50^Ti, respectively.
  In the other case that the instrumental mass bias may slightly differ from the exponential mass fractionation law, mass-independent Ti isotopic effects would be created from double-spike inversion and, therefore, a secondary normalization onto the bracketing OL-Ti standards would be necessary to obtain the correct ^50^Ti anomalies for unknown samples, in which a spline with the minimal mean squared weighted deviation value on the ε^50^Ti_prelim_ values of the OL-Ti standard can be used for the normalization:
  4 $${\varepsilon }^{50}{\rm{T}}{\rm{i}}={\varepsilon }^{50}{{\rm{T}}{\rm{i}}}_{{\rm{p}}{\rm{r}}{\rm{e}}{\rm{l}}{\rm{i}}{\rm{m}}-{\rm{s}}{\rm{a}}{\rm{m}}{\rm{p}}{\rm{l}}{\rm{e}}}-{\varepsilon }^{50}{{\rm{T}}{\rm{i}}}_{{\rm{O}}{\rm{L}}-{\rm{T}}{\rm{i}}{\rm{s}}{\rm{p}}{\rm{l}}{\rm{i}}{\rm{n}}{\rm{e}}}.$$
4. It is, however, notable that the primary double-spike inversion includes no correction of the ^46^Ti anomaly. Following equation (4), a ε^50^Ti value can be obtained for an unknown sample from averaging the results from duplicate measurements, after which a ε^46^Ti value can be further inferred on the basis of the correlation between ε^46^Ti and ε^50^Ti, that is, ε^46^Ti = (0.184 ± 0.007) × ε^50^Ti + (0.025 ± 0.009) (ref. ^71^). An ideal way to correct for the ^46^Ti anomaly is to create an equivalent effect on the standard composition before double-spike inversion:
  5 $${\left({{\rm{R}}}_{{\rm{standard}}}^{46/47}\right)}_{{\rm{new}}}={{\rm{R}}}_{{\rm{standard}}}^{46/47}\times \left(\frac{{{\rm{\varepsilon }}}^{46}{\rm{Ti}}}{\mathrm{10,000}}+1\right).$$
5. As the correction of the ^46^Ti anomaly would affect the calculated *λ*, *α* and *β* values from double-spike inversion and then the calculated ε^46^Ti and ε^50^Ti values, an iteration of procedures (1) to (4) needs to be carried out using the revised standard composition, and the ε^50^Ti values for unknown samples normally converge after four or five iterations. The preliminary mass-dependent Ti isotopic fractionations (reported as a delta notation on the ^49^Ti/^47^Ti ratio relative to the standard composition) can be obtained from *α*:

6 $${{\rm{\delta }}}^{49}{{\rm{Ti}}}_{{\rm{prelim}}}=\left({{\rm{e}}}^{-\alpha \times {\rm{ln}}\left({m}_{49}/{m}_{47}\right)}-1\right)\times \mathrm{1,000},$$

in which *m*_47_ and *m*_49_ stand for the atomic masses of ^47^Ti and ^49^Ti, respectively. In the case that the instrumental mass fractionation bias did not follow exactly an exponential mass fractionation law, a secondary normalization onto the bracketing OL-Ti standard is necessary to obtain the correct mass-dependent Ti isotopic fractionations for unknown samples, in which a spline with the minimal mean squared weighted deviation value on the δ^49^Ti_prelim_ values of the OL-Ti standard can be used for the normalization:

7 $${{\rm{\delta }}}^{49}{\rm{T}}{\rm{i}}={{\rm{\delta }}}^{49}{{\rm{T}}{\rm{i}}}_{{\rm{p}}{\rm{r}}{\rm{e}}{\rm{l}}{\rm{i}}{\rm{m}}-{\rm{s}}{\rm{a}}{\rm{m}}{\rm{p}}{\rm{l}}{\rm{e}}}-{{\rm{\delta }}}^{49}{{\rm{T}}{\rm{i}}}_{{\rm{O}}{\rm{L}}-{\rm{T}}{\rm{i}}{\rm{s}}{\rm{p}}{\rm{l}}{\rm{i}}{\rm{n}}{\rm{e}}}.$$

### Propagation of uncertainty from anomaly correction

The uncertainties from the derivation of ^46^Ti anomalies from the measured ^50^Ti anomalies and the subsequent correction need to be propagated onto the results. Main uncertainties on the derived ^46^Ti anomalies should come from (1) uncertainties on the ^50^Ti measurements and (2) uncertainties from the assumed relation between ε^46^Ti and ε^50^Ti, that is, ε^46^Ti = (0.184 ± 0.007) × ε^50^Ti + (0.025 ± 0.009). We consider that the 2 s.e. value of the ε^50^Ti_prelim_ values from duplicate measurements of each sample to represent the uncertainty on the ^50^Ti measurements for this sample, that is, *σ*(ε^50^Ti_prelim_). The uncertainty on the inferred ^46^Ti anomaly can be approximated to:

8 $$\sigma \left({{\rm{\varepsilon }}}^{46}{\rm{Ti}}\right)\approx \sqrt{{\left[\sigma \left({{\rm{\varepsilon }}}^{50}{{\rm{Ti}}}_{{\rm{prelim}}}\right)\times 0.184\right]}^{2}+{0.009}^{2}}.$$

The effects from ^46^Ti correction on the δ^49^Ti and ε^50^Ti values can be empirically evaluated by assigning various ε^46^Ti values for correction within the data-processing protocol described above, which follows linear equations of the assigned ε^46^Ti value:

9 $${{\rm{\delta }}}^{49}{{\rm{Ti}}}_{{\rm{corr}}}-{{\rm{\delta }}}^{49}{{\rm{Ti}}}_{{\rm{uncorr}}}\approx 0.108\times {{\rm{\varepsilon }}}^{46}{\rm{Ti}},$$

10 $${{\rm{\varepsilon }}}^{50}{{\rm{Ti}}}_{{\rm{corr}}}-{{\rm{\varepsilon }}}^{50}{{\rm{Ti}}}_{{\rm{uncorr}}}\approx -0.96\times {{\rm{\varepsilon }}}^{46}{\rm{Ti}}.$$

The uncertainty on the derived ε^46^Ti value from equation (8) can be further propagated onto the δ^49^Ti and ε^50^Ti results:

11 $$\sigma \left({{\rm{\delta }}}^{49}{\rm{Ti}}\right)\approx \sqrt{{\left[\sigma \left({{\rm{\varepsilon }}}^{46}{\rm{Ti}}\right)\times 0.108\right]}^{2}+{\left[\sigma \left({{\rm{\delta }}}^{49}{{\rm{Ti}}}_{{\rm{prelim}}}\right)\right]}^{2}},$$

12 $$\sigma \left({{\rm{\varepsilon }}}^{50}{\rm{Ti}}\right)\approx \sqrt{{\left[\sigma \left({{\rm{\varepsilon }}}^{46}{\rm{Ti}}\right)\times \left(-0.96\right)\right]}^{2}+{\left[\sigma \left({{\rm{\varepsilon }}}^{50}{{\rm{Ti}}}_{{\rm{prelim}}}\right)\right]}^{2}}.$$

Note that the pooled uncertainties on the ε^50^Ti_prelim_ and δ^49^Ti_prelim_ values from duplicate measurements are ±0.15 and ±0.010‰, respectively. Substituting these values into equations (8), (11) and (12) shows that the propagated uncertainties from anomaly correction are negligible relative to the uncertainties on ε^50^Ti_prelim_ and δ^49^Ti_prelim_.

### Results and data reproducibility

Although simulation shows that the use of a ^47^Ti–^49^Ti double spike provides optimally small errors on the results for a large spiking range (*f*_sample_ = 0.20–0.80, in which *f*_sample_ stands for the sample fraction in the sample–spike mixture^73^), in practice, there may be systematic offsets in the calculated δ^49^Ti value when acquiring data at different spiking ratios, for example, up to about 0.18‰ offsets for the spiked Ti Alfa Aesar aliquots that have *f*_sample_ values between 0.20 and 0.80 (ref. ^35^). Despite the magnitude of the offsets at different spiking ratios depending on the calibration of the standard composition and the used ^47^Ti–^49^Ti double spike in different laboratories, it is worthwhile scrutinizing the effects and, if necessary, optimizing the *f*_sample_ values between the samples and the bracketing standard. Except for the Cold Bokkeveld sample (*f*_sample_ = 0.470), all the samples in this study have *f*_sample_ values within a small range (0.409–0.454), which closely match that of the used bracketing OL-Ti standard solutions (*f*_sample_ = 0.43–0.44). Several runs of three reference materials (that is, BHVO-2, BCR-2 and AGV-2) and two chondrites (Murchison and Murray) at different spiking ratios show that, within a *f*_sample_ range of 0.409–0.454, no systematic offset relative to the bracketing OL-Ti standard (*f*_sample_ = 0.43–0.44) was resolved at a precision of ±0.15 for ε^50^Ti and of ±0.010‰ for δ^49^Ti.

**Extended Data Fig. 2:**
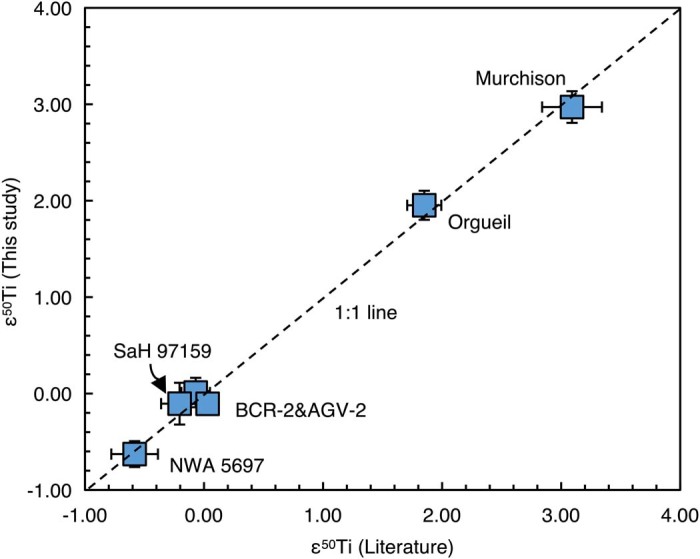
Comparing the measured ^50^Ti anomalies of bulk chondrites and terrestrial rocks with those from the literature. Chondrite samples Murchison, Orgueil, SaH 97159 and NWA 5697 are plotted on the basis of the reasonings that: (1) enstatite chondrites (for example, SaH 97159), Murchison and Orgueil were previously shown to have small variations in ^50^Ti anomaly among various digestion aliquots, therefore being suited to check the robustness of ^50^Ti measurements in this study, and (2) NWA 5697 in this study was digested with a large mass of powders (about 2 g), which should not be affected much by heterogeneity inside the sample. The ε^50^Ti values of SaH 97159 and NWA 5697 are compared with the mean values of the literature data for enstatite chondrites and ordinary chondrites^69,70^, respectively.

Several runs of reference materials BHVO-2, BCR-2 and AGV-2 provide δ^49^Ti values of +0.024 ± 0.010‰ (*n* = 9, 2 s.d.), +0.001 ± 0.006‰ (*n* = 8, 2 s.d.) and +0.097 ± 0.013‰ (*n* = 4, 2 s.d.), respectively. These are within uncertainty identical to the previously recommended values in the literature^3,4,32,34,35^. With respect to anomaly measurements, all of the duplicate runs of reference materials BHVO-2, BCR-2, AGV-2 and BIR-1 give a mean ε^50^Ti value of −0.07 ± 0.14 (*n* = 19, 2 s.d.). The consistency of the δ^49^Ti and ε^50^Ti values from several runs of the same samples suggests a long-term external precision of ±0.010‰ and ±0.15, respectively, on the δ^49^Ti and ε^50^Ti data from this study. It is also noteworthy that the ε^50^Ti values of both terrestrial reference materials and chondrite meteorites, including Murchison, Orgueil, NWA 5697 and SaH 97159, are consistent with the values acquired previously in refs. ^69,70^ using a non-spike method (Extended Data Fig. 2), which demonstrate that the ε^50^Ti results derived from double-spike measurements in this study are accurate at the claimed precision.

### Precise and accurate determination of the δ^49^Ti average for whole-rock chondrites

There is notable scatter of the δ^49^Ti data reported for whole-rock chondrites in the literature, for instance, a δ^49^Ti average of +0.008 ± 0.039‰ (*n* = 16, 2 s.d.) from Greber et al.^33^, of +0.071 ± 0.085‰ (*n* = 22, 2 s.d.) from Deng et al.^34^ and of +0.047 ± 0.071‰ (*n* = 6, 2 s.d.) from Williams et al.^35^. However, we note that large offsets in δ^49^Ti (up to 0.100‰) were observed between lithium metaborate fusion digestions of the same komatiite and eucrite powders (for example, 501-1, 501-8, M657, M663, M666, M712, Lakangaon and Ibitira; Extended Data Fig. 3a) in Greber et al.^33^, and the authors have ascribed the discrepancy to a lack of equilibration of the sample with the double spike that results in lower δ^49^Ti values^33^.

**Extended Data Fig. 3:**
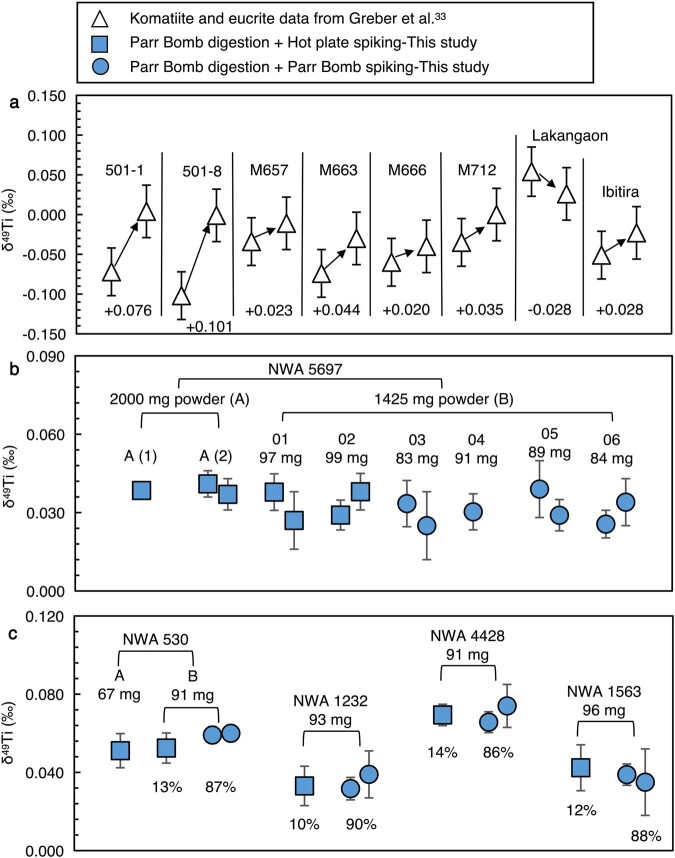
Comparing duplicate measurement results from this study with those from the literature. **a**, Duplicate measurement results of komatiite and eucrite samples in Greber et al.^33^. **b**,**c**, Experiments on whole-rock chondrites that involve two different spiking procedures: NWA 5697, NWA 530, NWA 1232, NWA 4428 and NWA 1563. Our analytical procedure has improved the data reproducibility by threefold to fivefold relative to those reported in Greber et al.^33^ (**a**).

For the digestion or spiking protocols involving HF acids, fluoride formation hampers either full-sample dissolution or sample–spike equilibration. Here we have carried out experiments to evaluate the potential effects from fluorides on the δ^49^Ti data in this study as follows:

1. An approximately 1,425-mg chip of NWA 5697 (L3) meteorite was crushed into a fine powder (NWA 5697-B) and six aliquots with masses of 83 to 99 mg (-01, -02, -03, -04, -05 and -06) were digested following the typical Parr bomb digestion procedure. Aliquots containing about 6 µg Ti were taken from ‘-1’ and ‘-2’ digestions and spiked in 6 M HCl on a hot plate at 120 °C, whereas the other four whole digestions were spiked and placed into a Parr bomb with 14 M HNO_3_ acids at 190 °C for a day, at which conditions fluorides should decompose. The six experiments provide consistent δ^49^Ti values (+0.032 ± 0.004‰, *n* = 6, 2 s.d.) that agree with the results from a roughly 2,000-mg digestion of NWA 5697 (-A) (+0.039 ± 0.001‰, *n* = 2, 2 s.d.) (Extended Data Fig. 3b). This confirms that the analytical protocol used in this study is sufficient to destroy potential fluorides formed from HF digestions.
2. The robustness of the protocol to eliminate fluorides can be further tested by a second set of experiments, in which fractions (12–14%) of the NWA 530, NWA 1232, NWA 4428 and NWA 1563 digestions were spiked and heated in 6 M HCl on a hot plate, whereas the remaining solutions were spiked and placed into a Parr bomb with 14 M HNO_3_ acids at 190 °C for a day. All four samples have identical δ^49^Ti values between the two procedures within an uncertainty of ±0.010‰ (Extended Data Fig. 3c).

**Extended Data Fig. 4:**
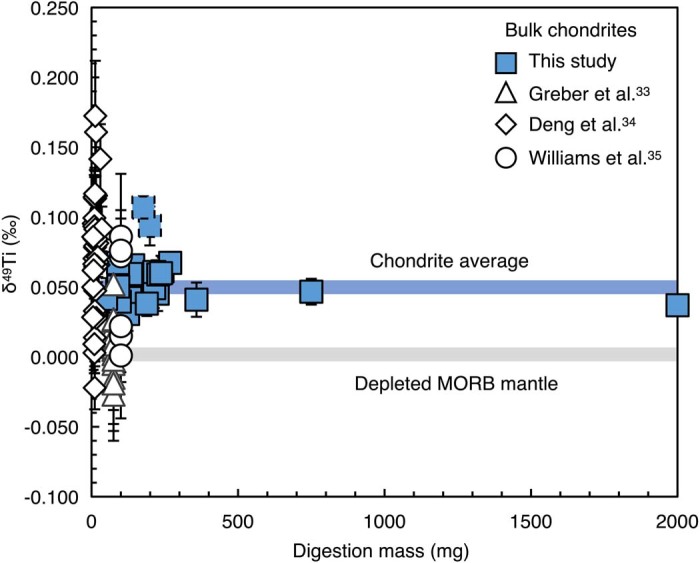
Comparing the δ^49^Ti data of whole-rock chondrites from this study and the literature with the digestion masses. The typical digestion masses for individual chondrite samples are roughly 50–100 mg in Greber et al.^33^, 5.3–35 mg in Deng et al.^34^ and ≥100 mg in Williams et al.^35^. The method in this study has improved the measurements of δ^49^Ti values for whole-rock chondrites by threefold compared with the previous data from ref. ^33^ (triangles), ref. ^34^ (diamonds) and ref. ^35^ (circles). The depleted MORB mantle value from refs. ^3,4^ is shown for comparison.

As heterogeneity does exist inside chondrites, for example, the large δ^49^Ti variation of −4‰ to +4‰ in Ca, Al-rich inclusions^71^, acquiring mass-dependent Ti isotope data for whole-rock chondrites can be subject to a certain degree of such heterogeneity. This can be well corroborated by the larger scatter in published δ^49^Ti data for whole-rock chondrites with the decreasing digestion masses (Extended Data Fig. 4). In this study, excluding Talbachat n’aït Isfoul (LL3) and NWA 2364 (CV3) that are probably subject to sample heterogeneity and show elevated δ^49^Ti values, the remaining 22 chondrite samples define an average δ^49^Ti of +0.053 ± 0.024‰ (2 s.d.) or ±0.005‰ (2 s.e.) (Extended Data Fig. 4). Our new chondrite average is identical to that of Deng et al.^34^ (+0.071 ± 0.085‰, *n* = 22, 2 s.d.) and Williams et al.^35^ (+0.047 ± 0.071‰, *n* = 6, 2 s.d.), but with a threefold improvement in precision. Considering the large digestion masses for most of the chondrite samples in this study, our new chondrite data should be least affected by sample heterogeneity. The new chondrite average is resolved to be around 0.052‰ higher than that of modern N-MORBs, that is, +0.001 ± 0.015‰ (2 s.d.) or ±0.004‰ (2 s.e.) (refs. ^3,4^) (Extended Data Fig. 4).

**Extended Data Fig. 5:**
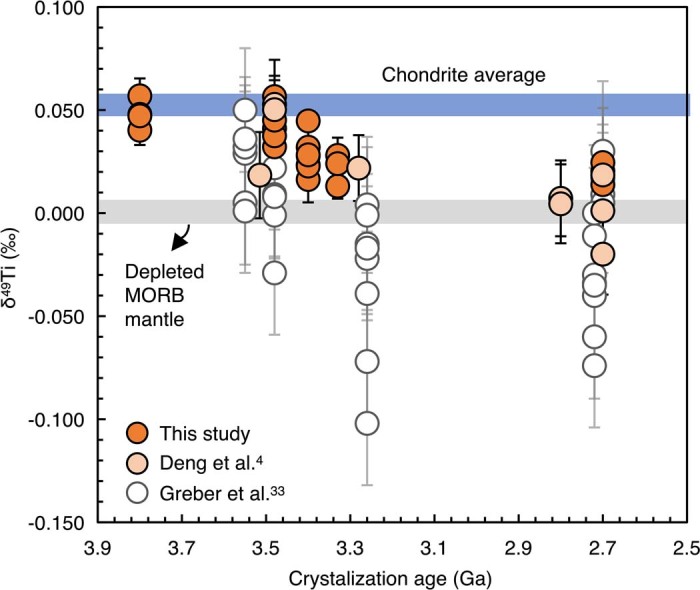
Comparing the δ^49^Ti data of Archaean mantle-derived rocks from this study (orange circles), ref. ^4^ (light orange circles) and ref. ^33^ (white circles). The δ^49^Ti averages of whole-rock chondrites from this study and the N-MORB samples from refs. ^3,4^ are also shown for comparison.

We note that data offset between laboratories also exists for the δ^49^Ti results from Archaean komatiites, with the substantially lower and more scattered δ^49^Ti values in Greber et al.^33^ than those in this study and Deng et al.^4^ (Extended Data Fig. 5). We emphasize that the presence of data discrepancy between digestion duplicates of the same komatiite powders in Greber et al.^33^ probably points to a larger analytical uncertainty on the reported δ^49^Ti dataset for both whole-rock chondrites and Archaean komatiites than the claimed precision of ±0.030–0.034‰ (95% confidence interval) for individual samples.

### Quantifying mass exchange between mantle and crustal reservoirs in deep time

Assuming that the continental crust (CC) at time *t*_*i*_ and the mantle equilibrated with the recycled crustal melting residues from continental crust formation (thereafter called the contaminated mantle, that is, CM) together form a primitive mantle (PM) reservoir with respect to TiO_2_ content and δ^49^Ti, the TiO_2_ fraction from continental crust in the CC-CM combination at time *t*_*i*_ (that is, ${{\rm{X}}}_{{{\rm{TiO}}}_{2}\_{\rm{CC}}\_{t}_{i}}$) should be:

13 $${{\rm{X}}}_{{{\rm{TiO}}}_{2}\_{\rm{CC}}\_{t}_{i}}=\frac{{{\rm{C}}}_{{{\rm{TiO}}}_{2}\_{\rm{CC}}}\times {q}_{{\rm{CC}}\_{t}_{i}}\times {m}_{{\rm{CC}}}}{{{\rm{C}}}_{{{\rm{TiO}}}_{2}\_{\rm{PM}}}\times \left({q}_{{\rm{CC}}\_{t}_{i}}\times {m}_{{\rm{CC}}}+{m}_{{\rm{CM}}}\right)},$$

in which ${{\rm{C}}}_{{{\rm{TiO}}}_{2}}$ represents the TiO_2_ content and *m* stands for the mass. We note that ${q}_{{\rm{CC}}\_{{\rm{t}}}_{i}}$ defines the fraction of the total continental crust (*m*_CC_) that has been produced until time *t*_*i*_, which has been provided in the continental crust growth models from refs. ^43,44^. The Ti isotopic composition of the contaminated mantle at time *t*_*i*_ should approximately follow:

14 $${{\rm{\delta }}}^{49}{{\rm{Ti}}}_{{\rm{CM}}\_{t}_{i}}=\frac{{{\rm{\delta }}}^{49}{{\rm{Ti}}}_{{\rm{PM}}}-{{\rm{\delta }}}^{49}{{\rm{Ti}}}_{{\rm{CC}}}\times {{\rm{X}}}_{{{\rm{TiO}}}_{2}\_{\rm{CC}}\_{t}_{i}}}{\left(1-{{\rm{X}}}_{{\rm{Ti}}{{\rm{O}}}_{2}\_{\rm{CC}}\_{t}_{i}}\right)}.$$

Assigning δ^49^Ti_PM_ = +0.053 ± 0.005‰ (this study) and the δ^49^Ti average of Archaean TTGs to be δ^49^Ti_CC_ (+0.381 ± 0.056‰, 2 s.e., *n* = 19; this study and refs. ^5,7^), ${{\rm{\delta }}}^{49}{{\rm{Ti}}}_{{\rm{CM}}\_{t}_{i}}$ is controlled by ${{\rm{X}}}_{{\rm{Ti}}{{\rm{O}}}_{2}\_{\rm{CC}}\_{t}_{i}}$. As ${{\rm{C}}}_{{{\rm{TiO}}}_{2}\_{\rm{PM}}}$ and ${{\rm{C}}}_{{{\rm{TiO}}}_{2}\_{\rm{CC}}}$ can be reasonably assumed to be 0.18 wt% and 0.34 wt%, respectively, ${{\rm{X}}}_{{\rm{Ti}}{{\rm{O}}}_{2}\_{\rm{CC}}\_{t}_{i}}$ is further related with two free parameters, that is, *m*_CC_ and *m*_CM_ in equation (13). Although modern continental crust is about 0.55% of the BSE in mass (that is, *m*_CC_modern_ = 0.0055 × *m*_BSE_), the total mass of continental crust (*m*_CC_) ever produced throughout the Earth’s history remains less clear. To quantify ${{\rm{\delta }}}^{49}{{\rm{Ti}}}_{{\rm{CM}}\_{t}_{i}}$, we can bring in two free parameters, that is, *k* describing the total mass of continental crust ever produced through the Earth’s history after a normalization to its modern mass (*k* = *m*_CC_/*m*_CC_modern_) and *f* representing the fraction of Earth’s mantle to equilibrate with the recycled melting residues, that is, *f* = (*m*_CC_ + *m*_CM_)/*m*_BSE_. By assuming *k* and *f*, we can obtain the evolution of δ^49^Ti_CM_ through time in Fig. 2 based on the continental crust growth models from refs. ^43,44^.

## Online content

Any methods, additional references, Nature Portfolio reporting summaries, source data, extended data, supplementary information, acknowledgements, peer review information; details of author contributions and competing interests; and statements of data and code availability are available at 10.1038/s41586-023-06304-0.

## Figures and Tables

**Extended Data Table 1:**
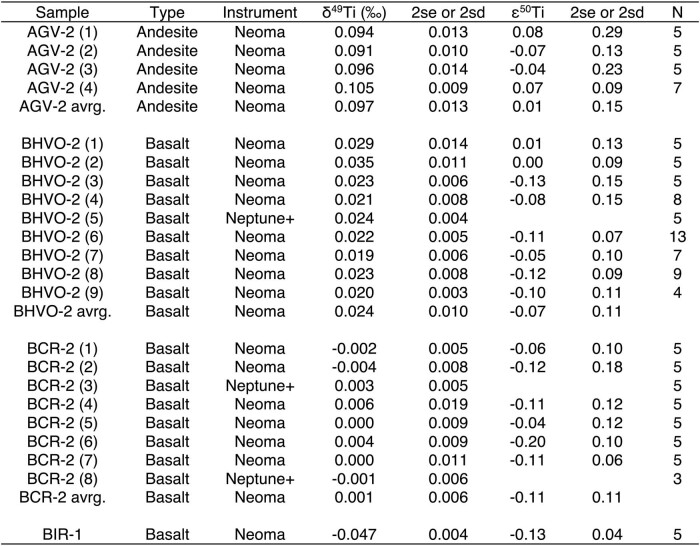
Titanium isotope results of terrestrial rock standards

**Extended Data Table 2:**
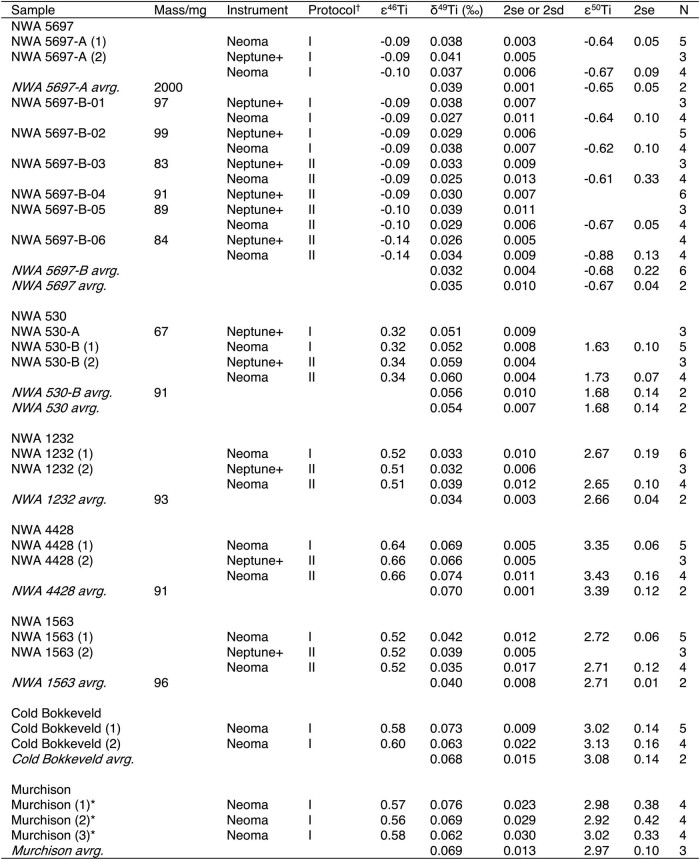
Results of experiments on whole-rock chondrites

*For the measurements of Murchison, each analysis contains only 50 cycles with an integration time of 8 s for each cycle, which explains the larger uncertainties relative to the results from other samples. †Protocol I: An aliquot containing approximately 6 µg Ti for each chondrite digestion was spiked and heated with 6 M HCl on a hot plate at 120 ^o^C. Protocol II: The whole digestions of whole-rock chondrites were spiked and placed into Parr Bomb with 14 M HNO_3_ acids at 190 ^o^C for one day.

**Extended Data Table 3:**
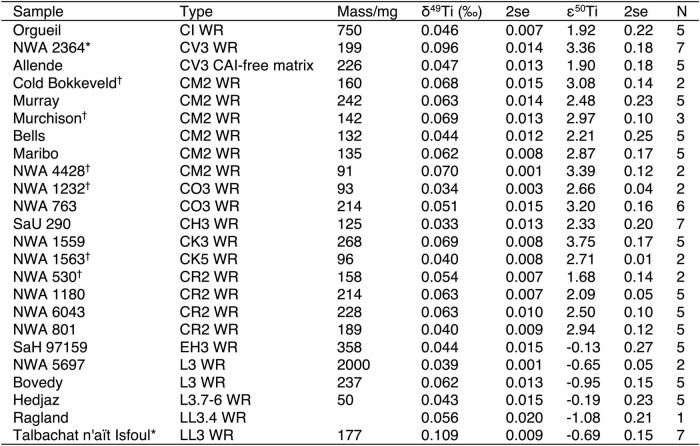
Titanium isotopic results of whole-rock chondrites

*The samples are not included in the calculation of chondrite average. †The average values of these samples have been calculated from several measurements in Extended Data Table 2.

**Extended Data Table 4:**
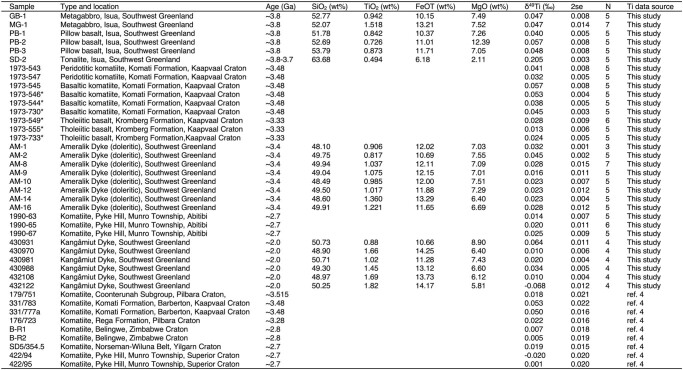
Chemical and Ti isotopic data of Archaean to Proterozoic samples from this study and the literature

Note that chemical data of the Isua samples are from ref. ^58^, and those of the Kangâmiut Dyke samples are from ref. ^75^. *These data have been measured using Neptune plus MC-ICP-MS following the conventional protocol.

**Extended Data Table 5:**
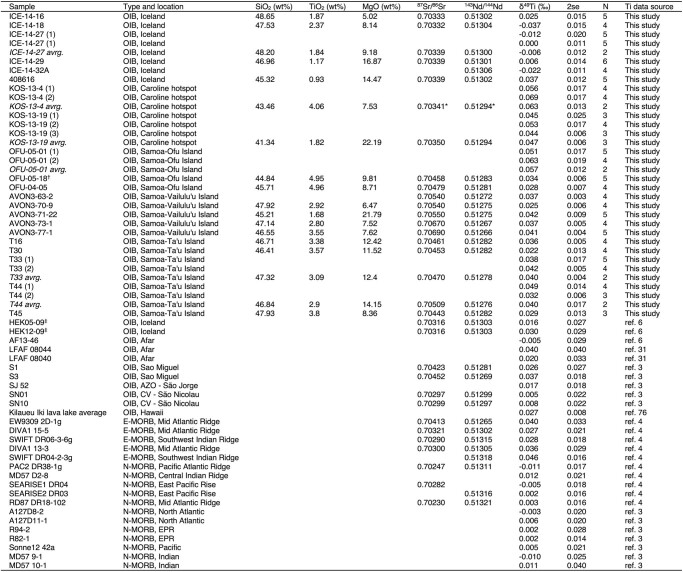
Chemical, Sr and Ti isotopic data of modern OIBs and MORBs

Note that chemical and Sr-Nd isotopic data of the studied OIB samples are from refs. ^55,64,65,66^. The δ^49^Ti average of Kilaueu Iki lava before Fe-Ti oxide fractionation is from ref. ^76^. *Representing the average value of the basalts from Caroline hotspot in ref. ^64^. †This sample was taken from the same dike as Ofu-04-06. ‡The Sr and Nd isotope data of the two samples are the average values calculated from data of Hekla basalts in ref. ^77^.

## Data Availability

All data are available at EarthChem^74^. Data supporting the findings of this study are provided with the paper (including Methods and Extended Data).
